# Systemic osteoarthritis: the difficulty of categorically naming a continuous condition

**DOI:** 10.1007/s40520-024-02714-w

**Published:** 2024-02-20

**Authors:** Gabriel Herrero-Beaumont, Francisco Castro-Dominguez, Alberto Migliore, Esperanza Naredo, Raquel Largo, Jean-Yves Reginster

**Affiliations:** 1 Bone and Joint Research Unit, Rheumatology Dept, IIS-Fundación Jiménez Díaz UAM, Madrid, Spain; 2 Rheumatology Department, Teknon Medical Center, Quironsalud Group, Barcelona, Spain; 3 Rheumatology Unit, San Pietro Fatebenefratelli Hospital, Rome, Italy; 4 WHO Collaborating Center for Epidemiology of Musculoskeletal Health and Aging, Division of Public Health, Epidemiology and Health Economics, University of Liège, Liège, Belgium

**Keywords:** Osteoarthritis, Systemic, Chronic inflammation, Metabolic syndrome, Personalized treatment

## Abstract

Osteoarthritis (OA) is a disease with systemic implications that go beyond joint problems. Its pathogenic mechanisms involve a variety of systemic conditions that contribute to joint damage. These include metabolic dysfunction, chronic low-grade inflammation, neuroplastic pain, and the influence of the central nervous system in the development of neuropathic pain. Besides, OA can negatively affect other aspects of health, such as quality of life, reduced physical activity, social isolation, depression, and anxiety. OA can be considered a complex system in which pathological interactions involve not only obesity and metabolic dysfunction, but also fragility syndrome, sarcopenia, neurological complications, and systemic energy redistribution. Complex systems are composed of multiple interacting and dynamic parts and exhibit emergent properties that cannot be fully explained by examining their individual components. Chronic low-grade inflammation is characteristic of OA, occurring both in the affected joint, and systemically, mainly due to adipose tissue inflammation in obese patients. Obesity is a key factor in the progression of OA, so primary treatment should focus on its control, while maintaining muscle health. The chronic inflammation could lead to changes in energy distribution among the affected joint tissues. Therefore, OA should be approached as a systemic disease, considering individual patient factors, such as genetics, inflammatory response, and lifestyle. Medical care should be more holistic and personalized. Consideration of a name change, such as "systemic OA", could help to move away from the perception of a disease focused only on the joints.

## Introduction

If hypocrisy is the price to be paid for immoral behavior, misguided labels often accompany a poor recognition of reality hindering desired progress. It is a saying that we should not attribute malice to what can be adequately explained by ignorance. However, is intentional ignorance justifiable? It is a fact that labeling a condition as osteoarthritis (OA), which is a progressive and continuous disorder characterized by a long pre-clinical stage, multiple risk factors that can follow various pathways, and a variety of manifestations and degrees of severity, has proven to be a significant challenge [1]. Conversely, diseases with discontinuous phenotypes present distinct and well-defined profiles of clinical expression; however, this is not the case in OA [2]. The diversity of symptoms, severity levels, and individual variations throughout the prolonged course of the disease, along with its associated risk factors, make it challenging to establish clear-cut boundaries or rigid classifications. In fact, quantitative genetics deals with the study of continuous phenotypes and posits the hypothesis that hundreds of genes are involved in phenotype variability. Each mutation only contributes a small effect to the expression of the disease [3, 4].

**Fig. 1:**
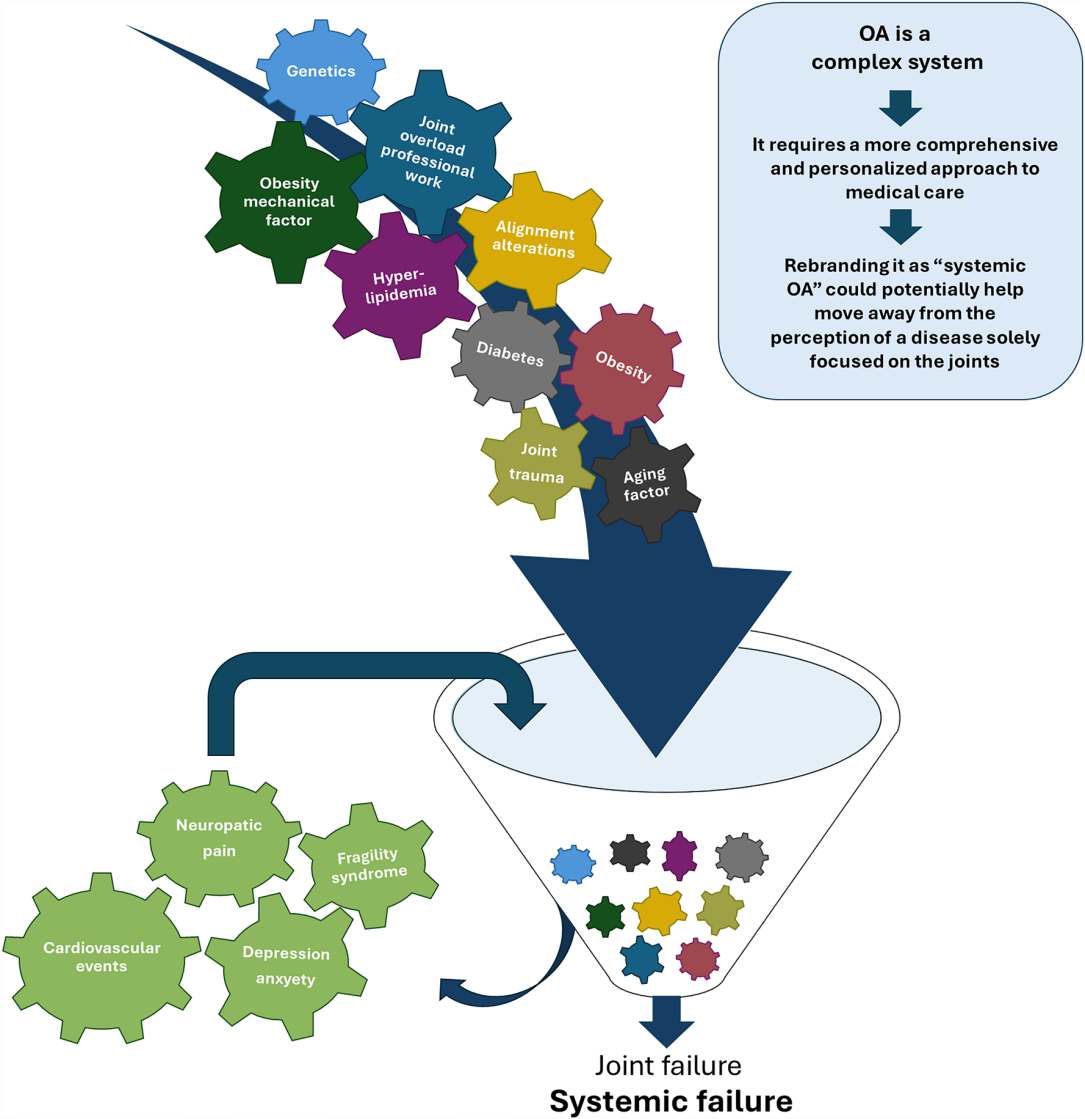
The intricacy of OA becomes more apparent when we relate it to the concept of a complex system. In fact, it can involve interwoven pathological interactions, encompassing not only obesity and related metabolic dysfunction but also frailty syndrome, sarcopenia, neurological complications, systemic energy redistribution, and others. These components and their interactions are in a constant state of flux. The whole exceeds the knowledge that we have of its parts. Moreover, when we analyze each element in isolation from the rest of the system, the inherent complexity of the entire system can dissipate, and the global entity disappears

In this sense, OA is a disease with broader systemic implications beyond just joint problems, since its pathogenic mechanisms involve various systemic conditions leading to articular damage. These include the components of metabolic syndrome, such as obesity, type 2 diabetes, hypercholesterolemia, and other conditions associated with obesity, alongside chronic low-grade inflammation [5–7], neuroplastic pain [8, 9], and the involvement of the central nervous system in the development of neuropathic pain (Fig. 1). OA shares common mechanisms with primary sarcopenia, and when both diseases coincide, the prognosis substantially worsens [10]. In addition, OA can also have systemic effects on other aspects of an individual’s health. For example, chronic pain and disability associated with OA can lead to decreased physical activity, social isolation, depression, or anxiety and decreased quality of life [11–13] (Fig. 1). All of this leads to an increase in cardiovascular-related morbidity and mortality [14–16]. Most of these risk factors also show a continuous, sometimes reversible spectrum.

The chronic inflammation characteristic of OA has been related to the activation of innate immunity in chondrocytes, which is driven by a robust activation of NF-κB, MAPK, and PI3K (phosphoinositide 3-kinase) pathways, resulting in significant production of pro-inflammatory cytokines, tissue-degrading enzymes, and inflammasome components [17]. It is well known that an excess of glucose and/or lipids, as well as the presence of microcrystals, contribute to this process [18, 19]. It is important to note that all of them converge on the same intracellular pro-inflammatory molecular signaling pathways [17]. In turn, the inflammatory process affects the synovium even before the degradation of the cartilage begins [20, 21]. Indeed, synovitis can manifest in the initial stages of OA and predict its development [21]. Finally, the osteochondral unit dynamically increases subchondral bone remodeling, resulting in sclerosis. Some authors suggest that in cases of generalized osteoporosis and hormonal changes during menopause, subchondral bone may experience an opposite effect, leading to subsequent subchondral bone osteoporosis, which can also accelerate OA progression [22]. In summary, while OA primarily affects the joints during its clinical course, complex pathological interactions involving systemic risk factors can occur even before the symptomatic phase.

At this point, it would be useful to differentiate between what is a complicated structure and a complex system, and then determine which concept best applies to OA. We could characterize a complicated structure as one in which there are multiple interrelated components that interact in a predictable manner and follow deterministic rules. In other words, the function is the strict sum of its parts [23]. However, there are phenomena that cannot be explained by their simple components. For instance, the basic molecule of water is H–O–H, but water unique properties emerge from different and changing interactions between groups of these molecules. The essence of water is H–O–H, but the quality of water is different from that of its molecule.

Likewise, we believe that OA cannot be fully explained by a single narrative or explanation, such as when it is described as a joint organ disease. The intricacy of the disease can be better understood by aligning it with the concept of a complex system. Indeed, it may encompass intricate pathological interactions, involving not only obesity and related metabolic dysfunction but also frailty syndrome, sarcopenia, neurological complications, and systemic energy redistribution (Fig. 1). A complex system can be defined as a set of elements that form a whole and carry out a function. The components and their interactions are dynamic. The whole exceeds the knowledge that we have of its parts. Complex systems, including biological systems, are composed of multiple interacting parts and exhibit emergent properties that cannot be fully explained by examining individual components in isolation [23]. Furthermore, analysis which studies each element by isolating it from the rest of the system causes the inherent complexity of the system to vanish, and the global entity disappears. Thus, although pre-clinical data suggest that very high cholesterol levels can directly worsen the progression of OA; some clinical studies in humans seems to suggest that the contribution of hypercholesterolemia to the progression of OA is not independent of the effect of obesity [24]. Consequently, when we attempt to develop categorical subgroups, and try to assess how much each component contributes to the overall system behavior, we could distort the essence of the disease. This phrase refers to the challenge of precisely describing a condition or state that exists on a continuous spectrum, using discrete or categorical terms.

Chronic inflammation of body fat tissue has been shown to be a key factor in the progression of OA. The elevated serum concentration of adipokines and other inflammatory mediators synthesized by the fat tissue in obese or overweight individuals is mainly responsible for the individual’s systemic inflammation [25, 26]. What is poorly described is whether body obesity is reflected in an increase of intra-synovial adipose tissue, or the effect of obesity in the inflammatory profile of intra-synovial adipocytes [27]. Indeed, the quantity and function of fatty tissue in the joint is still not fully understood [27].

Meanwhile, systemic and joint inflammation could lead to an intra-articular energy redistribution in the three tissues most affected by the disease: cartilage, synovium, and subchondral bone. Let us note that the nutrients for the cartilage are derived from subchondral and synovial vascularization. In this sense, it is possible that a redistribution of energy among the tissues may have a more detrimental impact on the cartilage, as the synovium becomes inflamed, consuming significant amounts of energy, and the subchondral bone undergoes a pro-anabolic sclerosis process. This particular situation does not occur in other organs of the body. In the cartilage, various metabolic pathways related to energy generation are altered. OA chondrocytes show mitochondrial impairment in which mitochondrial ATP generation is reduced, while the glycolytic pathway is activated [28–30]. In line with these data, different alterations in nutrient sensor pathways and glucose and insulin metabolism have been described in OA cartilage without fully understanding its consequences in an anaerobic cell such as the chondrocyte [29, 31–33]. Furthermore, the decrease in the expression of AMPK, a key enzyme in cellular ATP generation, was accompanied by an increase in its expression in the peripheral blood cells of the patients [29, 34]. These findings suggest that not only a shift in systemic energy distribution might intensify the cartilage damage, but there can also be a similar energetic distribution in the three joint tissues. Overall, it can be accurately stated that obesity impacts the progression of most diseases, although not uniformly [5]. In fact, for some of these conditions, the primary treatment approach is not typically centered on its management, as observed in RA. By contrast, in OA, the primary therapy should be focused on managing obesity and maintaining muscle health.

The basic element in OA is not the cartilage nor the joint, but the whole patient. Factors, such as the patient’s genetics related to pain expression, obesity, inflammatory response, bone metabolism, muscle strength, exercise, overloading activities, lifestyle, and environment, can all play a role in the development and progression of OA, and individual patient factors can influence the effectiveness of different treatments. By acknowledging the significance of a comprehensive patient-centered approach in the development and management of OA, healthcare providers can develop more personalized and effective treatment plans that address the individual needs and circumstances of each patient.

Undoubtedly, OA is essentially a systemic medical condition, treated by specialists who often do not assess the patient holistically. In this way, all patients in their earlier stages, as well as those with advanced OA deemed unsuitable for surgery, would receive a more accurate approach in rheumatology consultations. Meanwhile, patients exclusively linked to a mechanical/traumatic cause would derive greater benefit from an assessment conducted by orthopedic surgery. It is important to convey to our colleagues who are beginning their training the essential role that rheumatologists play in treating these patients. We must remind them that we are internists and not joint specialists.

Despite the diversity in the clinical expression of OA, recognizing it as a disease of continuous nature is crucial for providing personalized and effective healthcare. Would not it be time to consider a name change that would enable us to move away from the primarily joint-focused perception of the disease, and understand it as an essential addition to the obesity/metabolic-senescent framework? For example: systemic osteoarthritis.

## Data Availability

Data sharing is not applicable to this article.
